# The Effect of Gradient Bias Design on Electrochemistry and Tribology Behaviors of PVD CrN Film in a Simulative Marine Environment

**DOI:** 10.3390/ma11091753

**Published:** 2018-09-18

**Authors:** Shihong Cen, Xiaogai Lv, Beibei Xu, Ying Xu

**Affiliations:** 1 College of Pharmacy and Chemical Engineering, Zhengzhou University of Industrial Technology, Zhengzhou 451150, Henan, China; e13936613673@163.com (S.C.); zyzhj1314@163.com (X.L.); Mingxuanhansi1314@163.com (B.X.); 2 College of Chemistry and Chemical Engineering, Henan University, Kaifeng 475004, Henan, China

**Keywords:** CrN, bias voltage, electrochemistry, tribology, marine environment

## Abstract

CrN films with various bias voltage designs (−20, −50, −80, −20~−80 V gradient change) were prepared via arc ion plating. Scanning electron microscope (SEM), X-ray diffraction (XRD), nanoIndentor, electrochemistry workstation and tribo-meter were selected to evaluate the microstructure, mechanics, electrochemistry and tribology behaviors of as-prepared specimens in a simulative marine environment. By comparison, the adhesion force and anti-corrosion ability of CrN film with a gradient bias design were greatly enhanced compared with other films. The tribology behaviors of as-prepared specimens under various normal loads and sliding frequencies were deeply discussed. The result showed that the bias design played a critical role to impact the friction and wear behavior of film. Meanwhile, the CrN film with gradient bias design could bear a load of 25 N while other single CrN films failed, implying the strongest load-bearing capacity. Furthermore, at the same test condition, the lowest friction coefficient (COF) and wear loss were observed for CrN film with a gradient bias design, implying outstanding anti-friction and anti-wear abilities.

## 1. Introduction

With the rapid development of ocean shipping and marine resources exploration, all kinds of marine equipment face some serious tribological problems, which need to be addressed urgently [1,2,3]. For example, some pump valve pipes, hydraulic and power transmission systems are directly contacted by seawater, and their service life and safety largely depend on the tribological behaviors of system components [4,5]. Multi-arc ion plating technique is commonly used to prepare dense protective film, which can deposit solid materials in the forms of atoms or molecules onto substrate [5]. Among all films, CrN-based hard film has been widely used in many fields such as machining, metallurgy casting, decorative film and aerospace because of its high hardness, strong wear resistance, excellent chemical inertness and outstanding temperature oxidation resistance [6,7,8,9,10]. Xu et al. [11] explored the effect of deposition parameters on the composition, structures, density and topography of CrN films, and confirmed that the deposition parameters played a key role to affect the composition and structure of CrN film. Shan et al. [12] prepared the PVD CrN films with various N_2_ gas flows on steel and found that the hardness of these CrN films is about 18~27 GPa. Ye et al. [4] pointed out that the PVD CrN film presented a columnar structure and the critical load was up to 118 N. Meanwhile, a number of works have been reported regarding electrochemical tests of multiphase and dense structure CrN film in NaCl solution [13,14]. It was found that some interphase corrosion reactions would occur inside the film, which would change the current distribution on the surface of the film. Meanwhile, the change of current distribution could eliminate the aggregation of current in the micro-hole and greatly reduce the rate of pitting corrosion, eventually avoiding electrochemical corrosion damage in the membrane-based interface. Therefore, it is expected that CrN film will be deposited on the surface of marine equipment such as various types of spool, seat, gears and transmission components, effectively prolonging the service life of wearing parts and increasing the reliability of equipment operation.

Electrochemical corrosion is inevitable when the film is served in seawater. However, the corrosion reaction and reaction rate depend on the design of film composition and structure to a certain extent. Sproul and Hauert [15,16] pointed out that the multilayer, multicomponent, multiphase and isotropic nanocomposite films displayed a better overall performance than single-structure films. Meanwhile, Kok et al. [17] confirmed that the chemical composition of film was closely related to bias voltage. Warcholinski et al. [18] revealed that the properties of film were greatly affected by bias voltage, and similar results were found by many studies [19,20]. Although there has been extensive research into the effects of bias voltage change and structure design on the performance of CrN film in different mediums, the multilayer structure design of CrN film achieved by alternating bias voltage and its anti-corrosion and anti-wear abilities in simulative marine environments is rare. Hence, the CrN film under alternating bias voltage design (multilayer structure) will greatly enhance the corrosion and wear resistance in harsh conditions. In this paper, the friction and wear performances of four kinds of CrN films in seawater were investigated, and the relationships between the composition and corrosion resistance of as-prepared films were discussed.

## 2. Experimental

### 2.1. Deposition

CrN films were prepared on 316L steel (30 mm × 20 mm × 2 mm) and Si (100) wafer substrates by Flexicoat 850 Arc Ion Plating (AIP) system. Before deposition, the 316L stainless steels were firstly polished to 40 ± 5 nm (Ra). Later, all substrates (316L stainless steel and Si wafer) were first cleaned and then fastened at a pre-cleaned substrate holder in the deposition chamber. Afterwards, the reaction chamber was heated to 300 °C and background pressure was set to 3.00 × 10^−3^ Pa. The Ar gas (purity ≥ 99.99%) with flow rate of 100 sccm was added into deposition chamber. In the meantime, the Cr target (purity ≥ 99.5%) with target current of 65 A was opened. All samples were continuously bombarded at bias voltage of −800~−1100 V for 2 min. Then the Ar flow rate was increased by 350 sccm, the bias voltage was reduced by −20 V, a pure mental Cr transition layer (interlayer) was deposited for 30 min. Subsequently, the Ar flow was interrupted, the N_2_ gas (purity ≥ 99.99%) was set at 800 sccm, the vacuum level was controlled to 3.50 Pa, and the target current was kept at 65 A. However, the bias voltages of Cr target were −20, −50, −80, −20~−80 V alternating change, corresponding to the four different CrN films, which marked as C1, C2, C3 and C4 specimens, respectively. The deposition time of C1, C2 and C3 samples was 100 min. The alternating bias design of C4 specimen was −20 → −50 → −80 → −50 → −20 V and every section was 20 min.

### 2.2. Characterization

The FEI Quanta FEG 250 SEM was adopted to observe the cross-sectional morphology of specimens. The Bruker-AXS D8 Advance XRD was adopted to investigate the crystal structure of CrN films. The scanned area was 30°~80° and the scanning speed was 5°/min. The obtained spectrum was analyzed and the average grain size was calculated in this study. The grain size of film was calculated by the Scherrer equation:

Dc = K·λ/β·cos θ(1)

where Dc represents the average grain size (nm), K represents the Scherrer constant (K = 0.89), λ represents the X-ray wavelength, β represents the diffraction peak half-width and θ represents the Bragg angle (°).

The surface roughness (Ra) is investigated by Alpha-Step IQ profilometer. The process was completed six times and the result was the average value. MTS-Nano G200 nanometer press-fit test platform with a continuous stiffness method was chosen to obtain the hardness (H) and modulus (E) of as-prepared four kinds of CrN films on 316L substrate. Six different regions were selected for each sample and the pressed depth was about 1000 nm. These H values were obtained from the curves by the Oliver–Pharr method. CSM scratch tester was used to acquire the critical load between as-prepared specimens and 316L substrate. The PGSTAT302 Autolab workstation was built to characterize the electrochemical behaviors of these films in seawater. The artificial seawater was prepared according to American Standard ASTM D1141-98(2003), and the chemical composition is given in Table 1. At a temperature of 20 °C, the Pt plate was chosen for counter electrode, the saturated calomel electrode (SCE) was selected for reference electrode. The test area was kept at 10 mm × 10 mm, the corrosion current density (i_corr_) and corrosion potential (E_corr_) of films in seawater were obtained through Tafel curves. During Tafel test, the scan rate was 1 mV/s.

Friction and wear properties under different applied loads and frequencies in seawater were surveyed by pin-on-disc tribometer (UMT-3, CETR, Los Angeles, California, USA) in reciprocating mode. Because WC is a commonly used water-lubricating material, we chose a WC ball (94%WC + 6%Co, H = 14 GPa, E = 650 GPa) as the counterpart for simulating the actual working environment. The test temperature was 19 ± 3 °C and the humidity was 75 ± 5%. The diameter of WC counterpart was 3 mm and the test time was set at 30 min. To get the interaction between the test parameters (apply load and sliding speed) and tribology indices (COF and wear rate), the applied load was selected to 5, 15 and 25 N, frequencies of 1, 4 and 8 Hz were chosen. The cross-section profiles of wear tracks on the specimen after tribo-test were characterized by contact surface profiler (Alpha Step-IQ). The specific wear rate W (m^3^/Nm) of wear track was calculated by the subsequent equation:

W = ΔV/L * d(2)

where the area of cross-section multiplied by track length is the wear volume ΔV (m^3^), L (N) is the applied load and d (m) is the sliding distance during test process. Each group was tested three times (three parallel samples) under the same condition to collect an average value.

## 3. Results and Discussion

### 3.1. Microstructures

**Figure 1:**
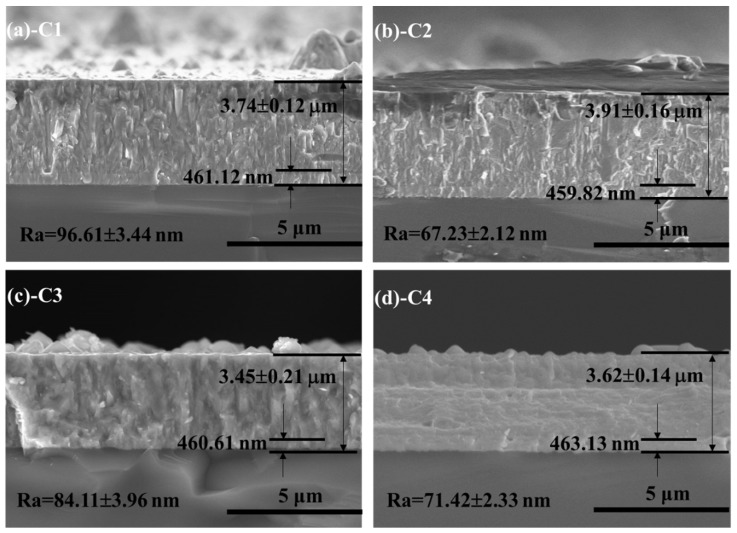
Cross-section morphology, thickness and surface roughness of all CrN films on Si (100) wafers: (**a**) C1; (**b**) C2; (**c**) C3; (**d**) C4.

Figure 1 shows the cross-section morphology, thickness and surface roughness of as-prepared specimens at different bias designs. As can be seen, the pure Cr interlayer is clear. The C1, C2 and C3 films present a typical columnar growth characteristic. However, the C4 film displayed a complex growth path and dense structure. The thickness and roughness have obvious differences, attributable to the change of bombarding energy under different bias voltages [21]. When the bias voltage increases in a small range (−20 to −50 V), the deposition rate (thickness) of film shows an upward trend. Nevertheless, because of the function of anti-sputtering, they are contrary when the bias voltage further increases (−50 to −80 V). In terms of surface roughness, the change rule is opposite; this is due to the fact that the surface large particles are easily shed by the bombardment of high energy particles and then leave micro-holes on the surface.

**Figure 2:**
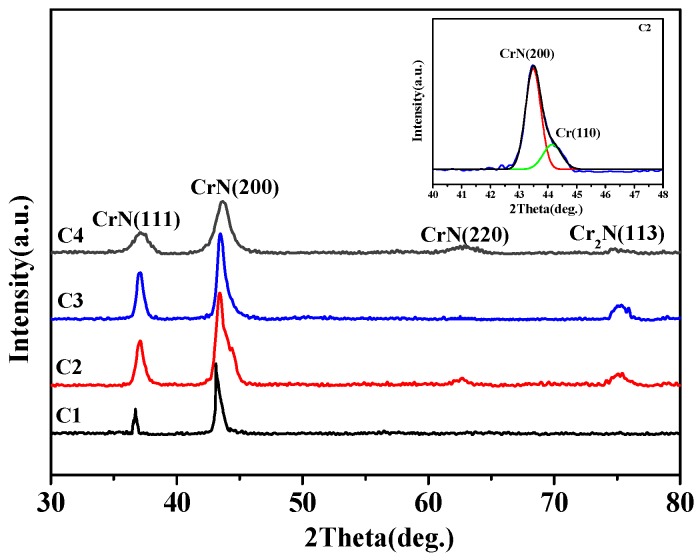
The XRD patterns of CrN films on 316L substrate.

Figure 2 shows the XRD patterns of four film samples. The main peaks exhibit a broadening trend along with the enhancement of bias voltage and the C4 sample presents the most apparently broadening phenomenon, indicating a decrease of grain size. In addition, the phase structure of films reveals an obvious change: CrN (C1) → CrN + Cr + Cr_2_N (C2) → CrN + Cr_2_N (C3), and the preferred orientation of CrN also change significantly. The negative bias voltage applied on the substrate during arc ion plating (AIP) process will exert a kinetic effect on the nucleation and growth stages of film, and then transform the microstructure and whole performances of film [22,23]. Hence, the deposition bias voltage will present a significant impact on the microstructure of film. When the bias voltage is −20 V, the CrN (111) and CrN (200) are sharp. Additionally, CrN (200) is the dominant growth direction. Nevertheless, the Cr phase is detected when the bias voltage increases to −50 V. Since the Cr (110) and CrN (200) of the C2 specimen are partially overlapped, the fitting peak of the overlapping region is shown in the top left of Figure 2. Meanwhile, the Cr (110) is vanished in the C3 sample, and the Cr2N (113) phase is found in the C2, C3 and C4 specimens. Some research [1,13,24] has pointed out that the Cr_2_N phase exhibited a better corrosion resistance in the NaCl solution than in the CrN phase. Thus, the phase structure is a key factor to measure the protective ability of film [25].

As shown in Table 2, the intensity ratio of (200) and (100) (I_(200)_/I_(111)_) value can reflect the change trend of preferential orientation. The large I_(200)_/I_(111)_ value indicates the strong preferential orientation of (200) crystallographic plane. With the increase of bias voltage, the growth tendency of (200) plane is significantly reduced. Combined with the preparation process of the C4 sample, the preferential growth direction of film possesses a gradient change. Although the C4 specimen shows a strong (200) orientation, the number of intergranular-voids and the average grain size are decreased significantly compared to other three films, which are shown in Figure 1 and Table 2. The preferred orientation is related to the wear resistance of film. Among all crystal phases of CrN film, Bull et al. [26] proved that the CrN film with the (200) oriented structure possessed the highest hardness and the best wear resistance. At the same time, some studies [27,28,29] reported that the reduction of grain size could significantly improve the mechanical properties (such as hardness), thereby enhancing the fracture stress. For C4 specimen, the pore path between the adjacent columnar grains will be complicated by the preferential orientation of gradient change, and could even block the corrosion channel of seawater.

### 3.2. Mechanical Performances

**Figure 3:**
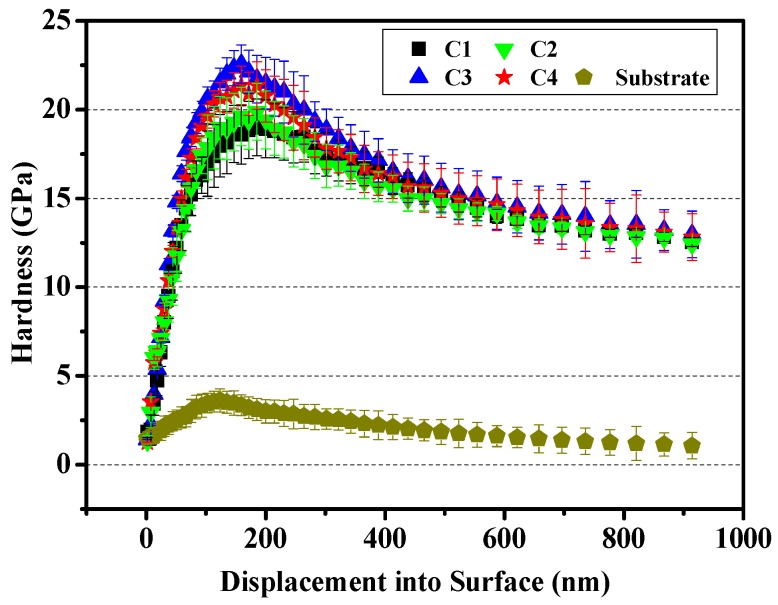
Nano indentation curves of 316L substrate and all films.

Hardness (H) and Elastic modulus (E) are two important parameters to inspect the mechanical properties of film. Generally, the enhancement of deposition bias voltage will improve the hardness and internal stress of film. The study result showed that the relationship between H and E of solid material depended on the energy dissipation capacity of material itself [30]. The smaller the ratio of H/E is, the smaller the elastic recovery of indenter becomes. Thus, the ratio of H/E can be more intuitive to reflect the toughness of film. Meanwhile, the H^3^/E^2^ value is a key parameter to measure the wear resistance of film, and a high H^3^/E^2^ ratio implies a strong ability of film to resist the crack propagation under external force [31]. The hardness, H/E and H^3^/E^2^ of substrate and CrN films are shown in Figure 3 and Table 3. It is clear that the hardness, H/E and H^3^/E^2^ of 316L substrate are about 6.9 GPa, 0.038 and 0.010 GPa, respectively, which are lower than CrN-coated samples. Among all CrN films, with the bias voltage increases from −20 to −80 V, the hardness, H/E and H^3^/E^2^ of CrN film show an ascend trend, which increase by 18.75%, 23.44% and 52.17%, respectively. In addition, the C4 specimen under alternating bias design of −20~−80 V presents a desirable hardness and toughness.

**Figure 4:**
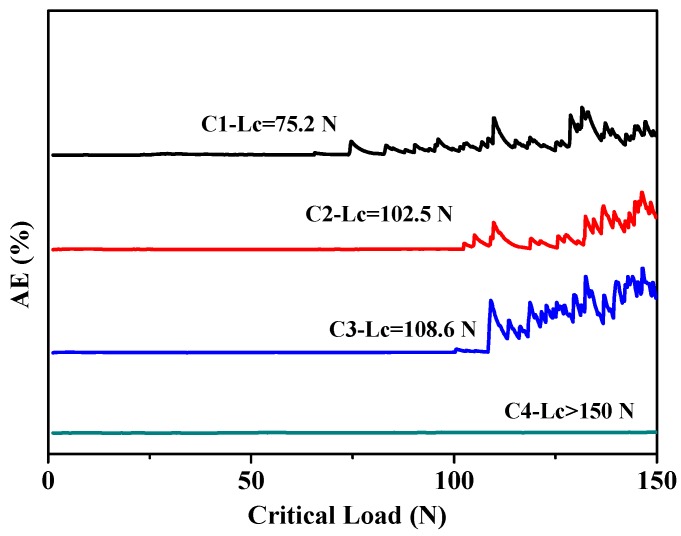
The acoustic signals and critical load of CrN films on 316L substrate.

**Figure 5:**
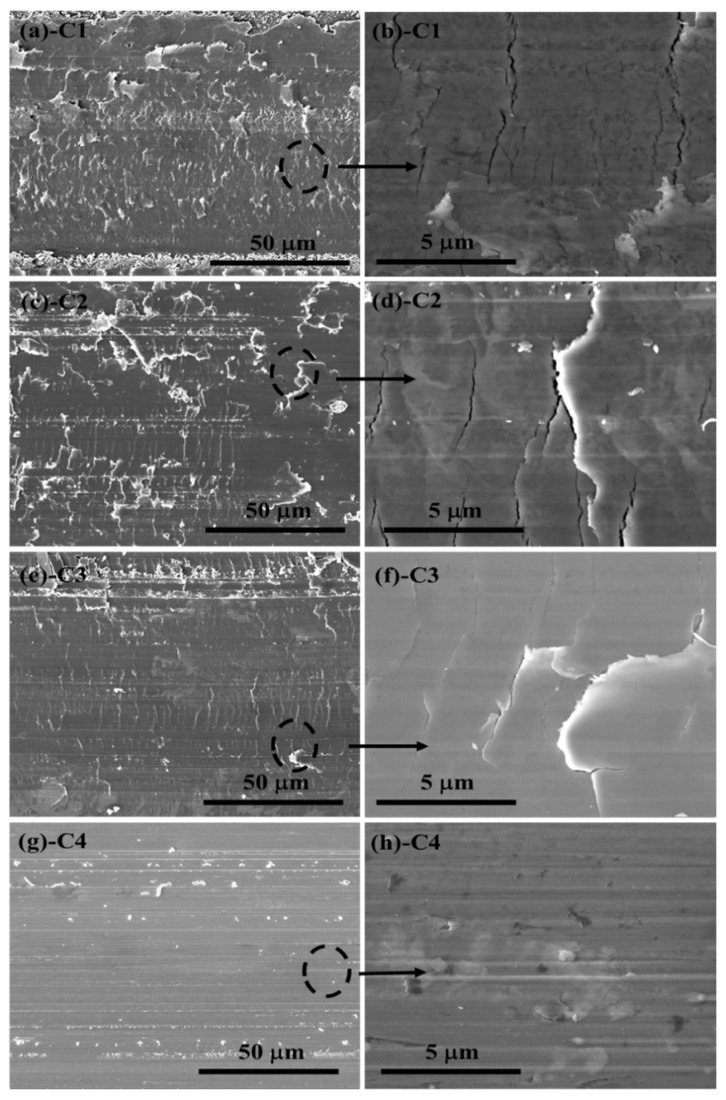
The SEM micrographs of local scratches for CrN films on 316L substrate: (**a**,**b**) C1; (**c**,**d**) C2; (**e**,**f**) C3; (**g**,**h**) C4.

The critical loads of as-prepared specimens are shown in Figure 4. It can be seen that the critical load of the C1 sample is about 75.2 N, which belongs to the range of high adhesion force [32]. With the increase of bias voltage, the acoustic signal corresponding to the critical load increases and exceeds 100 N. For the C4 specimen, the acoustic signal at the end of scratch has no significant fluctuation, that is, the critical load of the C4 sample is greater than 150 N. The SEM micrographs of local scratches are shown in Figure 5. When CrN film is prepared under a constant bias voltage, some radial cracks are observed in Figure 5a,c,e, which are perpendicular to the sliding direction, and the front crack is obviously bifurcation (feather area and atomization area). With the increase of bias voltage, the bifurcation ability of front micro-cracks is significantly inhibited, that is, the fracture stress σ is gradually increased. As shown in Figure 5e,g, there is no clear atomization area in the front crack, which indicates that the shielding effect for the front crack has a positive correction with the bias voltage. Combined with the data in Table 3, the hardness of film increases with the increase of bias voltage. Thus, the C4 specimen has a soft-hard alternating layer with varying hardness, which reduces the residual stress to a certain extent and improves the overall mechanical properties of film. As shown in Figure 5g,h, the C4 sample does not show any radial cracks at the end of scratch test. At the same time, it presents the shear-layer fault and block-slip failure mechanism, which is different to the fracture mechanism of other samples. For the rigid indenters with fixed geometries, the film will be deformed to accommodate the indentation volume when it is pressed by an indenter. Thus, the expansion of deformation zone will suffer an elastic constraints from internal film and then form a residual stress field. The stress field strength χ can be expressed as:

χ = ξθ(cot Φ)^2/3^(E/H)^1/2^ (3)

where Φ is the half cone angle of indenter, and ξθ is the deformation factor. A film with a low H/E ratio corresponds to a high residual stress field strength χ, so it is not easy to form the radial crack during the unloading stage [33]. In order to improve the critical load of film, an effective method involves suppressing the crack propagation in the inherent system, to increase the shielding effect of micro-crack front by the design of microstructure. The main way to achieve this is to optimize the complexity of microstructure, which can enhance the intergranular fracture stress σ and reduce the grain size. Finally, the micro-cracks can be suppressed in a narrow range.

### 3.3. Corrosion Performances

**Figure 6:**
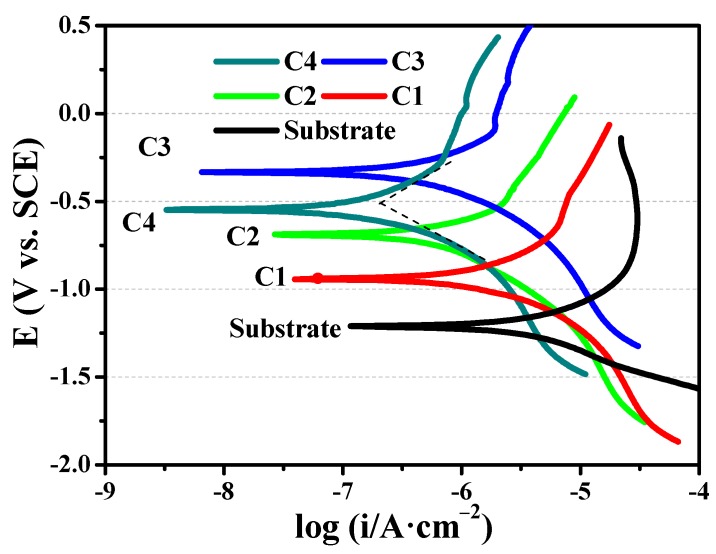
The polarization curves of 316L substrate and CrN films.

Metal materials often face crevice corrosion in seawater and the crevice of material surface will form the hypoxia zone. Because of the autocatalytic effect, Cl^−^ in seawater will be enriched in the crevice, so the corrosion rate of crevice is much larger than that of the material surface. The polarization curves of substrate and CrN films are shown in Figure 6, and the anodic slope (β_a_), cathodic slope (β_c_), corrosion current density (i_corr_) and corrosion potential (E_corr_) of CrN films in seawater are shown in Table 4. Clearly, the Tafel curve of 316L substrate is in the bottom right of the image and the i_corr_ of 316L substrate is about 5.11 × 10^−6^ A/cm^2^, which is higher than CrN films. After 316L substrate is covered by CrN film, the i_corr_ and E_corr_ produce a major improvement. With the increases of bias voltage from −20 to −80 V, the i_corr_ shows a downtrend and the E_corr_ presents an uptrend, manifesting that the anti-corrosion ability of C3 sample is stronger than C1 and C2 specimens. Moreover, the i_corr_ of C4 sample is only 3.82 × 10^−7^ A/cm^2^, which is 38.19% lower than C3 film (6.18 × 10^−7^ A/cm^2^). In other words, the corrosion rate of the C4 sample is the lowest in all specimens. In the meantime, when the bias voltage increases from −20 to −80 V, the β_a_ and β_c_ manifest an ascending trend, indicating that the polarization resistance is enhanced. Furthermore, the β_a_ and β_c_ of C4 film are higher than that of other films, revealing that the corrosion of anodic and cathodic are greatly suppressed. Combined with the microstructure analysis of C4 specimen (Figure 1 and Figure 2), the excellent electrochemical corrosion resistance can be attributed to the bias design of gradient change, which blocks the intergranular gaps of the longitudinal through-type. The formation of mixed phases (cubic CrN and trihedral Cr_2_N) and the presence of dense structure with fine grains are the critical factors to enhance the corrosion resistance of C4 sample. According to the literature [34,35,36], the gradient CrN film prepared by magnetron sputtering and arc ion plating methods showed an outstanding corrosion resistance in seawater, which was related to the gradient microstructure of film. Bertrand et al. [24] demonstrated that the Cr_2_N film exhibited a more excellent corrosion resistance than CrN film in acid and chloride solutions. Meanwhile, the corrosion resistance of CrN and Cr_2_N mixed phase film was stronger than that of single-phase CrN film.

### 3.4. Tribological Performances

**Figure 7:**
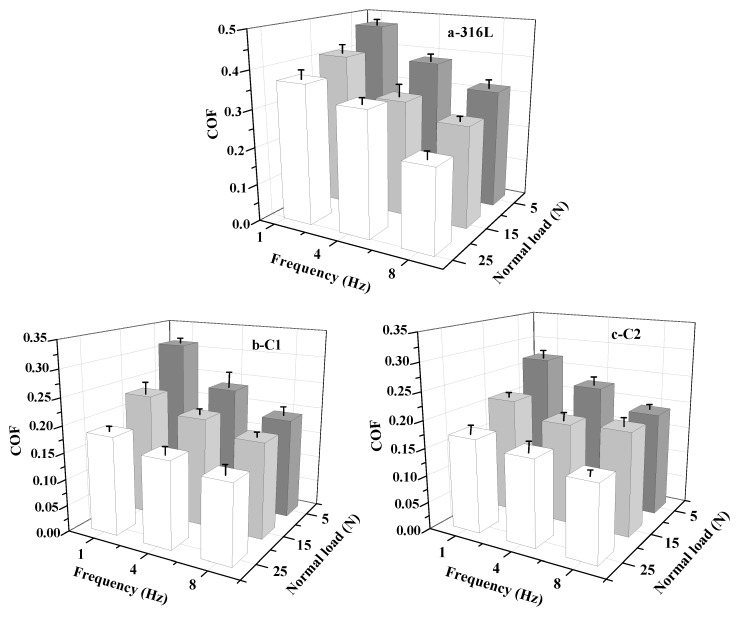

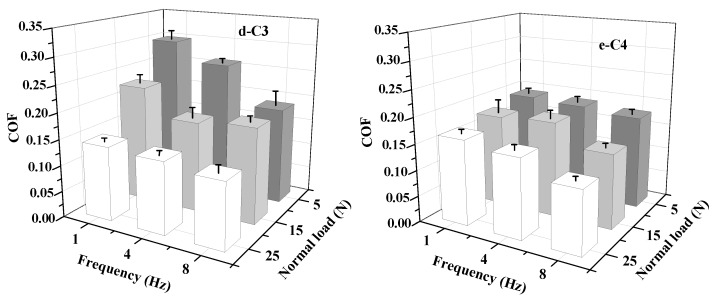
The COF of 316L substrate and CrN films in seawater: (**a**) 316L; (**b**) C1; (**c**) C2; (**d**) C3; (**e**) C4.

**Figure 8:**
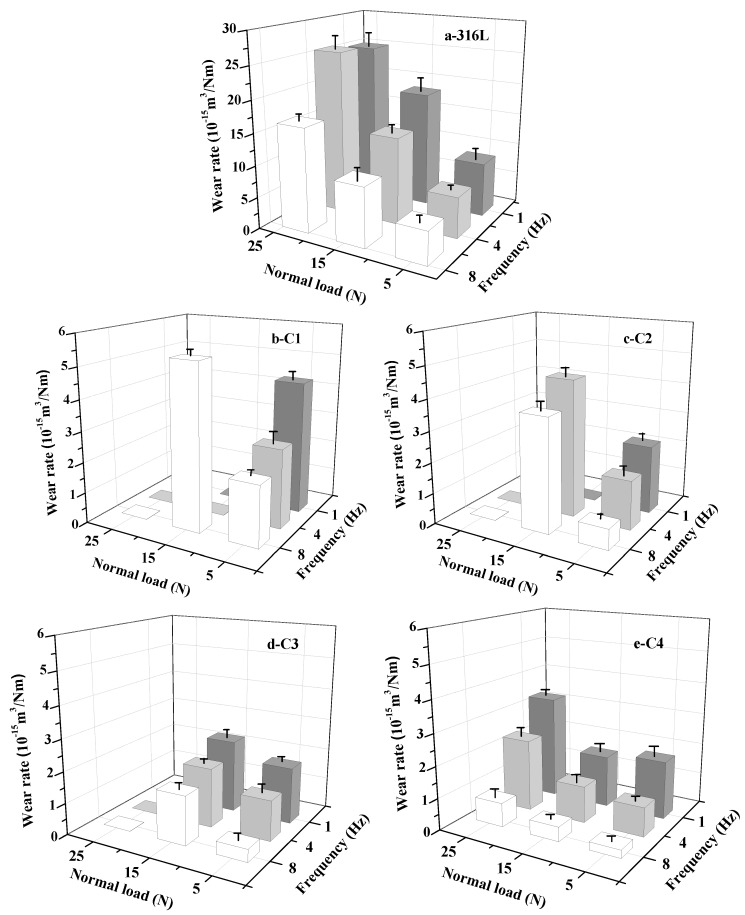
The wear rate of 316L substrate and CrN films in seawater: (**a**) 316L; (**b**) C1; (**c**) C2; (**d**) C3; (**e**) C4.

In order to understand the protective behavior and failure mechanism of as-prepared films in actual working conditions, the tribological behaviors must be discussed under the specific experimental environment and parameter setting. The friction pair, relative motion form, applied load and relative velocity are the influence factor of tribological behaviors [29]. Before discussion, we consider that the relative low hardness of WC counterpart displays a weak effect on friction and wear behaviors due to the small gap between the hardness of as-prepared CrN films. The COF and wear rate of substrate and all specimens are shown in Figure 7 and Figure 8. Since some specimens fail under certain conditions, the wear data of these experimental points cannot be calculated after wear testing. It is significant that the COFs of 316L substrate and CrN films show various characters under different friction conditions. The maximum value of 316L substrate (0.47) is found at the 5 N/1 Hz experimental point. The minimum value of C4 sample (0.11) appears at the 25 N/8 Hz experimental point. Nevertheless, the COFs under different applied loads and frequencies exert a good regularity. As a whole, the COF of these samples decreases with the increase of the slip velocity, and the COF at the low load point is higher than that at the high load point. However, the wear rate demonstrates a different change trend. The wear rate decreases with the increase of slip velocity, while the wear rate at the low load point is lower than that at the high load point. In this paper, the friction and wear behaviors of films are analyzed by lubrication medium of seawater, Hertzian contact stress, micro-bulge contact state and plastic deformation rate of film during running-in stage. Compared to pure water, some frictional chemical products with sludge-like compositions exhibit a certain degree of lubricating effect on the contact surfaces [37,38]. However, the corrosion in seawater cannot be neglected. Seawater can penetrate into the crack of friction zone, and the cracks are easy to expand under the action of friction and hydraulic pressure. On the one hand, it can accelerate the stripping of film. On the other hand, some localized electrochemical corrosion may occur in these cracks and then accelerate the film failure.

The occurrence of plastic deformation rather than peeling from substrate is a valid way to maintain the wear rate of film in a low range during the sliding [39]. Thus, the excellent hardness and toughness of film are the foundation for improving the wear resistance. The increase of film hardness can enhance the resistance of plastic deformation. Additionally, the improvement of toughness can effectively withstand the brittle fracture when the normal load is increased. As shown in Figure 3 and Figure 7, with the improvement of mechanical properties, the COF of film shows a downward trend.

For the normal load, the 316L substrate presents the highest COF under the low load condition (5 N, corresponding Hertzian contact stress of 1.33 GPa) and the highest wear rate at the high load point (25 N, corresponding Hertzian contact stress of 2.29 GPa). After the deposition of CrN films, the COF and wear rate are significantly reduced, while the C1 specimen fails at the condition of 15 N/8 Hz. This is attributed to its relatively low hardness and toughness compared with other samples, which is prone to occur during plastic deformation and even during the wear process. Furthermore, the larger grain size in film cannot effectively prevent the expansion of cracks, which are caused by the combination function of micro-cutting and fatigue wear. Due to the presence of seawater, debris (hard debris generated by film peeling) in the film-counterpart contact zone can be expelled in time and does not induce severe abrasive wear. However, seawater can penetrate into the film along the intergranular space, which can sharply reduce the service life of film under the interaction of wear and corrosion. The result is in agreement with the friction and wear data under the condition of high load (25 N, the corresponding Hertzian contact stress is 2.86~3.18 GPa), although the critical loads of C1, C2 and C3 samples belong to the high level, while these samples are entirely ineffective within 30 min due to the osmotic corrosion of seawater. It can be seen that the single improvement of critical load between film and substrate cannot satisfy the requirements of its service in seawater. In addition, the anti-corrosion ability of C3 and C4 specimens display a great difference in spite of the similar mechanical performances. The complex mixed crystal phase and fine grain structure of C4 sample result in the drastic reduction of infiltration capacity of seawater. That is to say, it can slow down the corrosion rate in seawater and highlight the lubrication effect of seawater, eventually prolonging the failure time of film. Thus, the C4 specimen does not fail in 25 N/8 Hz condition (contact stress 3.07 GPa) and emerges with a relatively low-wear rate.

**Figure 9:**
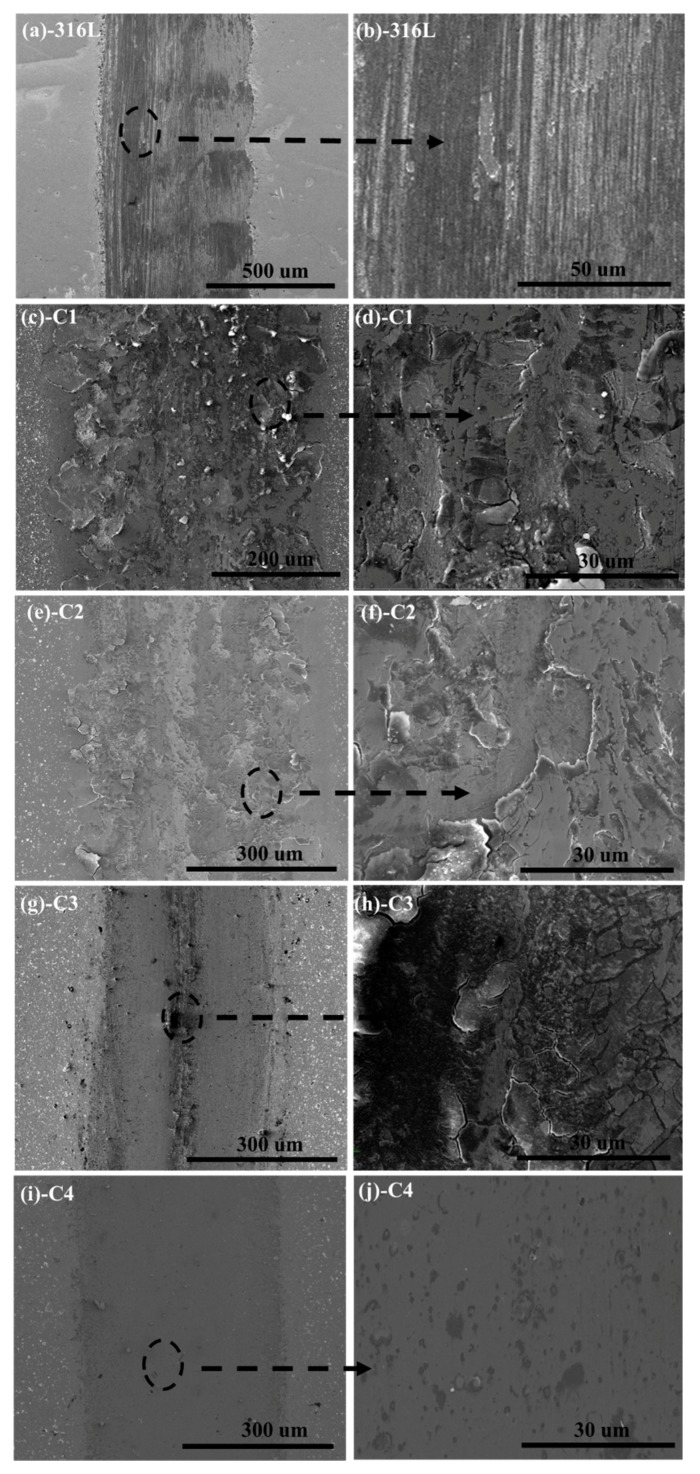
The morphologies of wear track on 316L substrate and CrN films: (**a**,**b**) 316L; (**c**,**d**) C1; (**e**,**f**) C2; (**g**,**h**) C3; (**i**,**j**) C4.

In order to further understand the actual wear situation of demarcation point, the local wear morphologies of substrate and CrN films after wear test are shown in Figure 9. Some obvious plastic deformation may be observed on the wear morphology of 316L substrate at 25 N/8 Hz (contact stress 2.29 GPa) (Figure 9a). By partial magnification, typical scratch and flaking are distributed on the contact surface (Figure 9b). As can be seen in Figure 9c–f, the C1 sample at 15N/8Hz (contact stress 2.41 GPa) experiment point and the C2 specimen at the 15 N/4 Hz (contact stress 2.56 GPa) experiment point emerge with a similar wear feature, which contains serious adhesion, crack and peeling behaviors. When the bias voltage further increases, the crack tip morphology of C3 sample at 15 N/1 Hz (contact stress 2.69 GPa) can be found in Figure 9g,h. It is easy to understand that seawater can penetrate into the film through the corrosion channel (crack and peeling) and induce the localized corrosion. Then the crack continues to develop and expand in the dual function of friction and hydraulic, eventually leading to the failure of film. Figure 9i,j show the wear track of C4 specimen in the 25 N/8 Hz (contact stress 3.07 GPa). Unlike substrate and other CrN films, the C4 sample can block the seepage of seawater because of the dense structure and the improvement of mechanical properties. These superior features can lead to the peeling failure caused by the extension of cracks being avoided.

**Figure 10:**
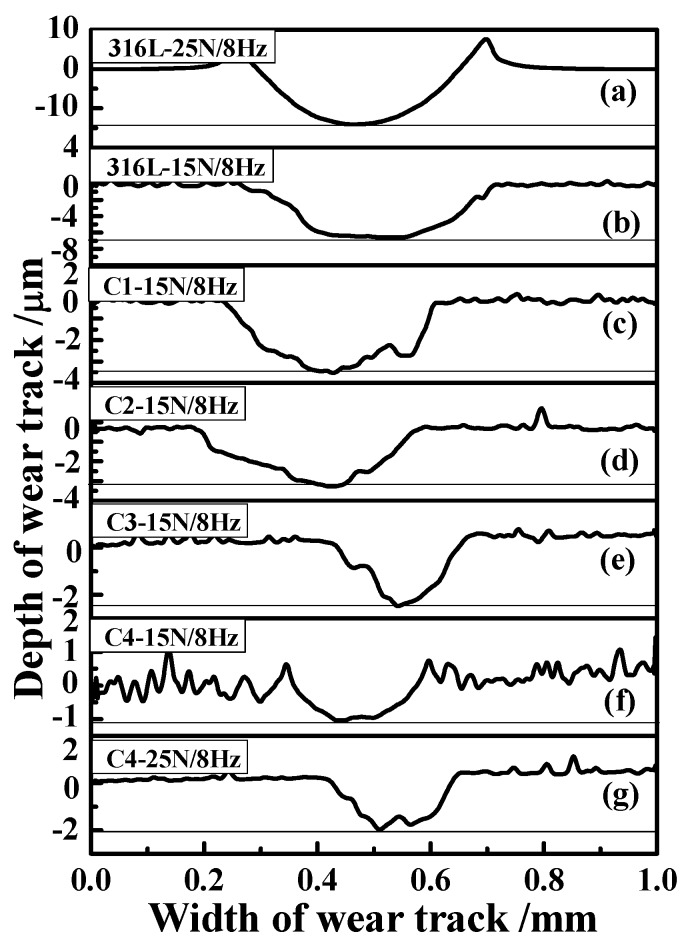
The cross-sectional profiles of wear track on 316L substrate and CrN films: (**a**,**b**) 316L; (**c**) C1; (**d**) C2; (**e**) C3; (**f**,**g**) C4.

**Figure 11:**
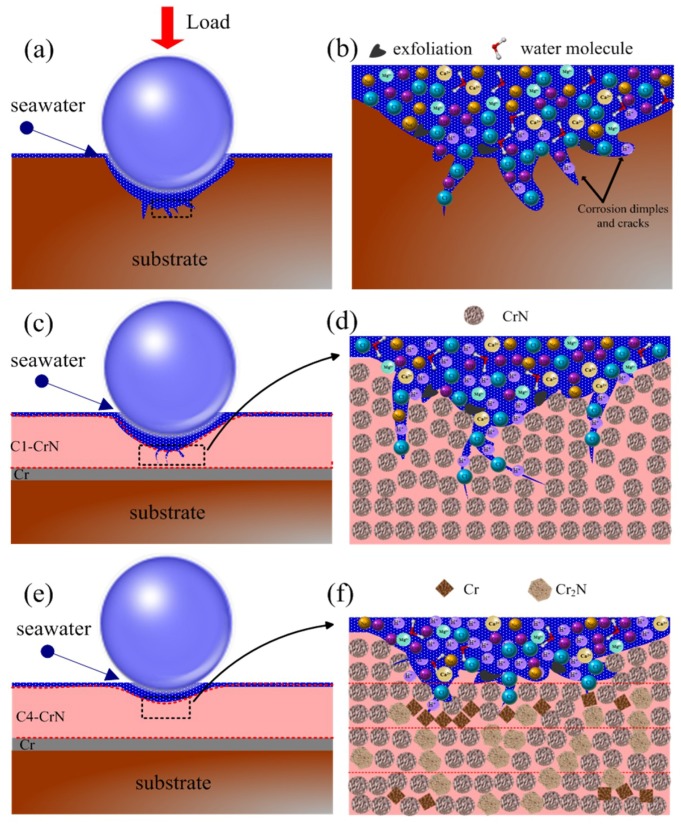
The detailed schematic diagrams of 316L substrate (**a**,**b**), C1 (**c**,**d**) and C4 (**e**,**f**) films in seawater.

In the meantime, the wear contours of demarcation point on substrate and CrN films are shown in Figure 10. For substrate and C4 film, the wear depth at 25 N/8 Hz is 15 and 2 μm, respectively, which is higher than that at 15 N/8 Hz. At the condition of 15 N/8 Hz, the depth of wear track on 316L substrate reaches to 7 μm, which is greatly higher than CrN-coated specimens. In detail, the wear depth of C1, C2, C3 and C4 samples is about 3.5, 3.1, 2.2 and 1.1 μm, respectively, which is 50.00%, 55.71%, 68.57% and 84.28% lower than that of substrate, respectively. Clearly, due to the design of alternating bias, the C4 specimen demonstrates the lowest depth and the best anti-bearing capacity, which are well in agreement with the results of the wear rate. Through the above analysis, the detailed schematic diagrams of substrate, single and multilayer CrN films are listed in Figure 11. Since the monolayer film exhibits a similar behavior, the C1 sample is chosen as a representative for analysis. For the 316L substrate, some peelings and corrosion dimples are preferentially formed on the wear track due to a lack of effectively protect film, low hardness and poor toughness (Figure 11a,b). During friction and wear tests, some passive films may be formed on the surface of the substrate. Nevertheless, the passive film may easily be destroyed through reciprocal sliding, which leads to the exposure of fresh surface in corrosive solution and then increasing wear-loss. For the C1 specimen, some micro-cracks are observed on the contact interface, which more easily form the penetrative channels under high load and high frequency (Figure 11c,d). These channels will promote the permeation of water molecule and some ions (Cl^−^, Na^+^, H^+^, etc.) through micro-cracks. Concurrently, the high concentration of Cl- ions can easily strip the surface oxide, resulting in the exposure of new surface to seawater. Besides, seawater can stimulate the new surface and then aggravate the wear, inducing more corrosion channels and forming a vicious cycle. Finally, the corrosion and wear loss are aggravated. Importantly, some slight micro-cracks and micro-holes are inevitably formed under extreme conditions, although the C4 sample possesses an excellent comprehensive performance (Figure 11e,f). However, sophisticated intergranular boundaries are formed by laddered changing bias design, which can effectively inhibit the growth of micro-cracks and micro-holes, and then the corrosion channels are preventable. Thus, the C4 specimen emerges with the best corrosion and tribological performances in seawater.

## 4. Conclusions

CrN specimens under various bias voltage designs were carefully obtained through the multi-arc ion plating technique. As the bias voltage increased (−20 → −50 → −80 V), the hardness of CrN film exhibited an uptrend. The relationships between bias design and tribology performance of CrN specimens were systematically examined in simulative marine environment. The multilayer CrN film presented better mechanical properties than individual CrN film. In the electrochemistry measurement, the i_corr_ of gradient CrN film was considerably lower than those monolayer CrN films due to its complex diffusion path. In the friction and wear test, the high hardness and gradient preferential orientation of CrN film had a forceful inhibitory effect on the generation and extension of crack under external force, which could efficaciously restrain the infiltration of seawater. Hence, the CrN film with gradient structure design (−20~−80 V) showed a lower COF and wear rate than a single CrN layer (−20, −50, −80 V) under high load and frequency conditions, indicating a good potential to improve the anti-wear and anti-corrosion abilities of pump valve pipes, and hydraulic and power transmission systems in marine environments.

## Figures and Tables

**Table 1:** Chemical composition of standard seawater.

Solution	NaCl	Na_2_SO_4_	MgCl_2_	CaCl_2_	SrCl_2_	KCl	NaHCO_3_	KBr	H_3_BO_3_	NaF
**Concentration**	24.53	4.09	5.20	1.16	0.025	0.695	0.201	0.101	0.027	0.003

**Table 2:** The intensity ratio of (200) and (100) (I_(200)_/I_(111)_) and average grain size of CrN films.

Film	I_(200)_/I_(111)_	Average Grain Size (nm)
C1	3.27	198 ± 18
C2	2.05	132 ± 11
C3	1.83	142 ± 16
C4	2.38	79 ± 9

**Table 3:** The hardness, H/E and H^3^/E^2^ of 316L substrate and CrN films.

Sample	H (GPa)	H/E	H^3^/E^2^ (GPa)
316L	3.6 ± 0.7	0.020	0.001
C1	18.2 ± 1.5	0.049	0.044
C2	19.7 ± 1.6	0.057	0.064
C3	22.4 ± 1.1	0.064	0.092
C4	21.5 ± 1.2	0.063	0.085

**Table 4:** The anodic slope (β_a_), cathodic slope (β_c_), corrosion current density (i_corr_) and corrosion potential (E_corr_) of 316L substrate and CrN films in seawater.

Sample	β_a_ (V dec^−1^)	β_c_ (V dec^−1^)	i_corr_ (A/cm^2^)	E_corr_ (V vs. SCE)
316L	0.36	−0.28	5.11 × 10^−6^	−1.24
C1	0.42	−0.32	9.96 × 10^−7^	−0.93
C2	0.46	−0.39	7.42 × 10^−7^	−0.72
C3	0.53	−0.45	6.18 × 10^−7^	−0.31
C4	0.83	−0.67	3.82 × 10^−7^	−0.55
